# Influence of Binasal and Uninasal Inhalations of Essential Oil of* Abies koreana* Twigs on Electroencephalographic Activity of Human

**DOI:** 10.1155/2016/9250935

**Published:** 2016-11-30

**Authors:** Min Seo, Kandhasamy Sowndhararajan, Songmun Kim

**Affiliations:** ^1^Department of Biological Environment, Kangwon National University, Chuncheon, Gangwon-do 24341, Republic of Korea; ^2^Gangwon Perfume Alchemy Co., Ltd., Chuncheon, Gangwon-do 24341, Republic of Korea

## Abstract

*Objectives*. The present work investigates the effect of essential oil from the twigs of* Abies koreana* on electroencephalographic (EEG) activity of human brain in order to understand the influence of binasal and uninasal inhalations.* Methods*. To accomplish this study, the essential oil from the twigs of* A. koreana* (AEO) was isolated by steam distillation and the EEG readings were recorded using QEEG-8 system from 8 grounding electrodes according to the International 10-20 System.* Results*. D-Limonene (25.29%), bornyl acetate (19.31%), camphene (12.48%), *α*-pinene (11.88%), *β*-pinene (6.45%), and eudesm-7(11)-en-ol (5.38%) were the major components in the essential oil. In the EEG study, the absolute alpha (left frontal and right parietal) and absolute fast alpha (right parietal) values significantly increased during the binasal inhalation of AEO. In the uninasal inhalation, absolute beta and theta values decreased significantly, especially in the right frontal and left and right parietal regions. The results revealed that the AEO produced different EEG power spectrum changes according to the nostril difference.* Conclusion*. The changes in EEG values due to the inhalation of AEO may contribute to the enhancement of relaxation (binasal inhalation) and alertness/attention (right uninasal inhalation) states of brain which could be used in aromatherapy treatments.

## 1. Introduction

Essential oils are complex mixture of volatile components including terpenes (mainly mono- and sesquiterpenes) and their oxygenated derivatives, which produce characteristic fragrance [1]. The essential oils have been used in the aromatherapy treatment throughout the world since ancient times due to their characteristic fragrance with the presence of various bioactive components. Particularly, fragrances of essential oils affect the psychophysiological conditions of human [2]. Previous studies have reported that the inhaled essential oils produce various positive psychological effects such as reducing stress and enhancing relaxation and alertness states of human brain function [3–5]. Although essential oils are used for many conditions, experimental studies on psychopharmacological properties of inhaled essential oils on human are very few.

It was reported that, due to slight turbinate swelling in one nostril, the air flow is greater into one nostril than into another nostril. The nostril that takes in more air moves from the left to the right one and back again every few hours, but the effect of this moving on the perception of smell has not been clear. In addition, many persons have an uneven septum, which makes one nostril larger than the other [6, 7]. Hence, it is possible that both or individual nostrils may produce different effects on brain function during the exposure of fragrance. The psychological changes stimulated through fragrance exposure are mainly related to the modulation of olfactory nervous system and subsequent alteration of the neuronal activity [8, 9]. It is well known that human electroencephalograph (EEG) activity is susceptible to alteration during exposure to fragrance. Therefore, the EEG is a good neurophysiological assessment tool to reflect the brain state or brain function [10].

The genus* Abies* (Pinaceae) comprises 51 species and is widely distributed in temperate and boreal regions of the northern hemisphere, mainly in mountainous regions [11]. In traditional systems of medicine, the different species of* Abies* have been used to treat various ailments such as colds, stomach ache, indigestion, and pulmonary diseases [12]. Among these species,* Abies koreana* Wilson (Korean fir; Korean name: Kusang namu) is a slow growing shrub or broadly pyramidal evergreen tree and widely occurs in the high mountain areas of Republic of Korea including Mt Dukyu, Mt Chiri, and Mt Halla [13]. Several lignans and triterpenoids (secocycloartenoid and two lanostane-type) have been isolated from this plant [14–16]. Previously, some authors reported the essential oil composition of* A. koreana* and its antimicrobial activity [12, 13, 17, 18]. Kim et al. [19] reported that the supercritical carbon dioxide fluid extract from the needles of* A. koreana* showed memory enhancing effect in mice. To the best of our knowledge, there is no published report on the fragrance stimulation effect of* A. koreana* essential oil. Based on the above knowledge, the current study was carried out to investigate the effect of inhalation of essential oil from the twigs of* A. koreana* (AEO) on electroencephalographic (EEG) activity of human brain with special reference to influence of binasal and uninasal inhalations.

## 2. Materials and Methods

### 2.1. Plant Material

The twigs of* A. koreana *were collected from Inje, Gangwon Province, Republic of Korea, in April 2015. The plant was authenticated and deposited in the Herbarium, Daejin University, Pocheon, Gyeonggi-do, Republic of Korea, with voucher number DJU-20152382.

### 2.2. Steam Distillation Extraction

The essential oil was extracted by steam distillation for 90 min using a Clevenger-type apparatus. The steam distillation was carried out with 1 kg of fresh* A. koreana* twigs. The extracted essential oil was dried by using anhydrous sodium sulfate and was then stored at 4°C until tested. The yield of essential oil was determined in triplicate.

### 2.3. GC-MS Analysis of AEO

GC-MS analysis was performed with a Varian CP3800 gas chromatography equipped with a VF‐5MS polydimethylsiloxane capillary column (30 m× 0.25 mm × 0.25 *μ*m) and a Varian 1200L mass detector (Varian, CA, USA). Helium was used as a carrier gas at the rate of 1 mL/min. Oven temperature was kept at 50°C for 5 min initially and then raised with rate of 5°C min to 250°C. The injected volume of essential oil was 10 *μ*L with a split ratio of 1 : 10. The injector temperature was set at 250°C. The mass spectra were recorded in the electrospray ionization mode at 70 eV in a scan range of 50–600* m/z*. The components of the AEO were identified by comparing the retention indices of the GC peaks obtained using homologous series of* n*-alkanes (C_8_–C_20_) with those reported in the literature [20]. The mass spectra of the peaks were also matched with standards reported in the literature and National Institute of Standards and Technology (NIST, 3.0) library.

### 2.4. EEG Study

The study followed the Declaration of Helsinki on Biomedical Research Involving Human Subjects and was approved by the ethics committee from the Kangwon National University Hospital, Chuncheon, Republic of Korea.

#### 2.4.1. Subjects

Twenty right-handed healthy volunteers (10 men and 10 women) aged between 20 and 30 years participated in this study. All the subjects are students and no one refused to participate in this study. None of the subjects had olfactory diseases, smoked, or abused drugs. All subjects gave their informed consent before participation.

#### 2.4.2. Experimental Design

A single group pretest and posttest experimental design was used in this study (20 subjects). A careful measurement was carried out before and during the inhalation of essential oil. Prior to experiment, the subjects were screened for an olfactory evaluation test by using the commercial perfumes. The subjects were told that the purpose of the study was to determine the effect of inhalation of essential oil on EEG activity. The subjects were instructed to sit quietly, close their eyes, and breathe normally during the measurement. After the EEG recordings, the subjects were asked to give their preference and impression of the fragrance of AEO. Further, none of the participants indicated that they felt that the essential oil had affected them in any way.

#### 2.4.3. EEG Recordings

The EEG readings were recorded using QEEG-8 system (LXE3208, LAXTHA Inc., Daejeon, Republic of Korea). The silver/silver chloride electrodes were placed on the scalp at left prefrontal (Fp1), right prefrontal (Fp2), left frontal (F3), right frontal (F4), left temporal (T3), right temporal (T4), left parietal (P3), and right parietal (P4) according to the International 10-20 System. The ipsilateral earlobe electrodes were used as reference electrodes. The EEG sampling rate of the measured subjects was 256 Hz, filtered in the range of 0.5–50 Hz, and the readings were stored in a computer by the 12-bit AD conversion. The ECI electrode gel (Electro-Gel™, Electro-Cap International Inc., Eaton, OH, USA) was applied into an each electrode to connect with the surface of the scalp in order to reduce the electric resistance of the scalp below 5 kΩ.

#### 2.4.4. Fragrance Administration

The AEO was used as the fragrance stimulus. The stimulus was presented to the subjects in a randomized sequence. The EEG recording room was maintained with a constant temperature (23°C) and humidity (50%). The undiluted AEO (10 *μ*L) was added on the perfumer's paper strip and then placed about 3 cm in front of the subject's nose. EEG was recorded 45 s before and 45 s during the exposure of AEO. To understand the influence of binasal and uninasal inhalations of AEO, the EEG readings were recorded separately for binasal as well as uninasal inhalations before and during the fragrance exposure. The baseline EEG readings were recorded at eye-closed state for each condition. During the uninasal EEG recordings, one nostril was completely blocked by using the cotton. The interval time between each condition [binasal and uninasal (left and right nostrils)] was 3 min.

#### 2.4.5. Data Analysis

The mean power values [microvolt square (*μ*V^2^)] were calculated for 25 EEG analysis indicators (Table 1). The* t*-mapping of EEG waves of brain was constructed by using Telescan software package (LXSMD61, LAXTHA Inc., Daejeon, Republic of Korea). The SPSS statistical package 18 (SPSS, Inc., Chicago, IL, USA) was used for data analysis on EEG activity before and during the exposure of AEO by a paired Student's* t*-test based on the EEG power spectrum values (nasal as well as gender differences). The *P* value < 0.05 was considered significant and the values are expressed as the mean ± SEM.

## 3. Results 

### 3.1. Chemical Composition of Essential Oil from* A. koreana* Twigs

The essential oil obtained from the twigs of* A. koreana* was pale yellow in color with an intensely coniferous, green, and woody aroma and its yield was 0.77% (v/w) by steam distillation. The result obtained by the GC-MS analysis of AEO is presented in Table 2. In total, 23 compounds were identified based on the retention indices and mass spectral data in the essential oil, which accounted for 98.52% of the total oil. The components are listed in order of their elution from a VF-5MS column. The AEO contains 13 hydrocarbons (8 monoterpenes and 4 sesquiterpenes) and 10 oxygen-containing components. The most abundant classes were monoterpenes (60.93%) followed by oxygenated monoterpenes (25.24%) and sesquiterpene hydrocarbons (8.10%). D-Limonene (25.29%), bornyl acetate (19.31%), camphene (12.48%), *α*-pinene (11.88%), *β*-pinene (6.45%), and eudesm-7(11)-en-ol (5.38%) were the major components of the essential oil. In addition, the AEO contains an unsaturated terpene, santene (1.71%).

### 3.2. Effect of Binasal and Uninasal Inhalations of AEO on EEG Activity 

#### 3.2.1. Binasal Inhalation

**Figure 1:**
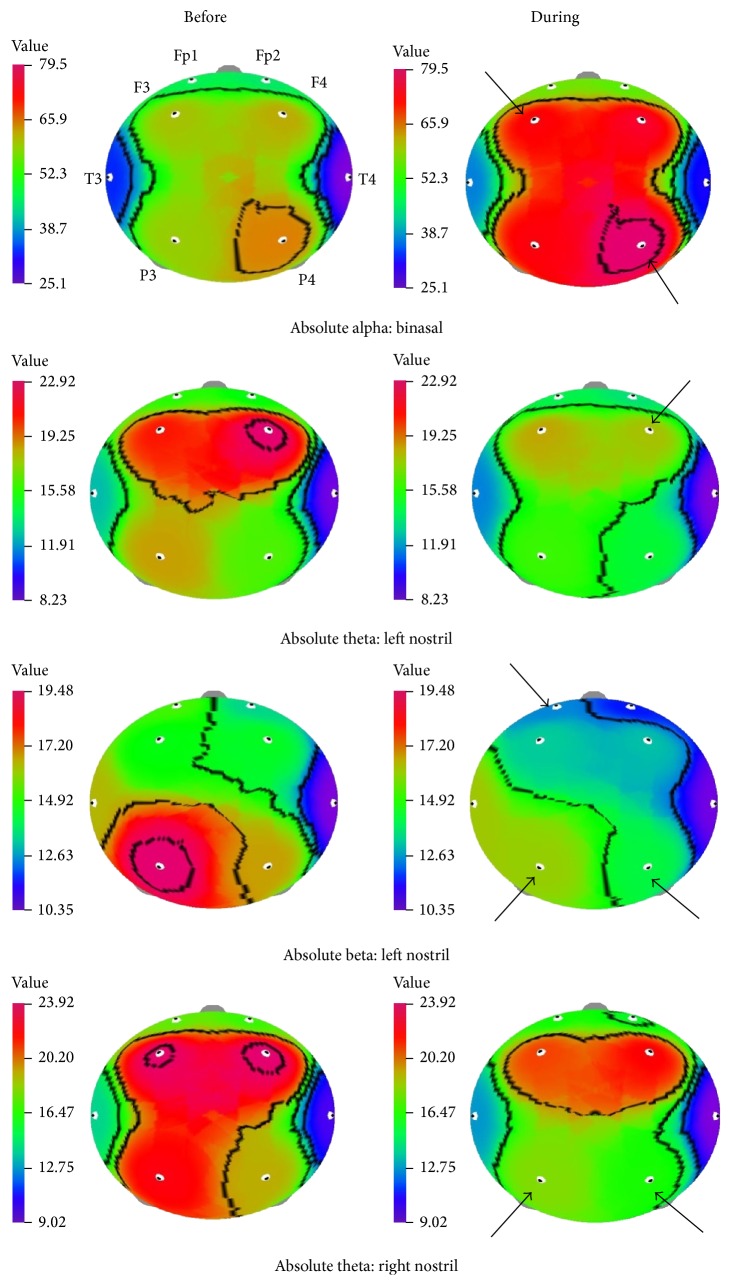
*t*-Mapping of EEG power spectrum changes before and during the binasal and uninasal inhalations of AEO. Fp1, left prefrontal; Fp2, right prefrontal; F3, left frontal; F4, right frontal; T3, left temporal; T4, right temporal; P3, left parietal; P4, right parietal. Arrows are showing significant changes in the regions during the inhalation of AEO.

The significant changes of EEG power spectrum values before and during the binasal inhalation of AEO are presented in Table 3. The EEG power spectrum values changed significantly due to the fragrance inhalation of AEO when compared with before inhalation. Out of 25 EEG indices, significant changes were detected in 7 indices during the binasal inhalation of AEO.  Figure 1 shows the* t*-mapping of significant changes of absolute power spectrum values. The EEG values of absolute alpha and mid beta significantly increased in left frontal (61.112–74.125 *μ*V^2^ and 4.927–6.141 *μ*V^2^, resp.) and right parietal (66.517–79.508 *μ*V^2^ and 5.261–6.833 *μ*V^2^, resp.) regions during the binasal inhalation AEO, whereas, absolute fast alpha value significantly increased only in the right parietal region (15.023–20.437 *μ*V^2^). The EEG changes were mostly occurred in the left frontal and right parietal regions than other regions. Further, relative mid beta in right parietal region significantly increased during the binasal inhalation of AEO.

#### 3.2.2. Uninasal: Left Nostril

The EEG power spectrum changes before and during the uninasal inhalation of AEO through left nostril are presented in Table 4. The results reveal that the AEO produced significant changes in 6 indices through left nostril inhalation. The absolute theta (right frontal) and beta including low beta (left parietal), mid beta (right and left parietal), and high beta (right frontal and right and left parietal regions) values significantly decreased during the inhalation of AEO through left nostril. The* t*-mapping also reflects the changes of absolute theta and beta waves (Figure 1). Among them, absolute beta values significantly decreased in three regions such as left prefrontal (15.552–12.738 *μ*V^2^), right (16.967–14.437 *μ*V^2^), and left parietal (19.483–16.379 *μ*V^2^) regions. In contrary to binasal inhalation, the absolute mid beta values significantly decreased from 7.086 to 5.822 *μ*V^2^ in the right parietal region.

#### 3.2.3. Uninasal: Right Nostril

Table 5 shows the significant changes of EEG power spectrum values during the uninasal inhalation of AEO through right nostril. Significant changes of EEG power spectrum values were observed only in 2 indices during the inhalation of AEO through right nostril.  Figure 1 shows the* t*-mapping of absolute theta wave before and during the inhalation. The absolute theta in right (19.540–16.896 *μ*V^2^) and left parietal (22.468–18.520 *μ*V^2^) regions and relative theta in right parietal region (0.178–0.158 *μ*V^2^) significantly decreased during the inhalation of AEO through right nostril.

### 3.3. Effect of Inhalation of AEO on EEG Activity: Gender Difference

The EEG power spectrum values significantly changed during the inhalation of AEO according to gender variation. The changes in absolute and relative changes of EEG activities were presented in Tables 6 and 7. The absolute fast alpha activity change in the right parietal region was similar in both gender and women during the binasal inhalation of AEO. In uninasal inhalation, similar changes of absolute beta (left parietal) and absolute high beta (left parietal) were observed in both gender and women through left nostril inhalation. On the other hand, similar changes of absolute low beta (left parietal) and absolute theta (right parietal) were observed in both gender and men during the uninasal inhalation of AEO through left and right nostrils, respectively. In general, the EEG power spectrum values highly affect women during both the binasal and uninasal inhalations of AEO when compared with men.

## 4. Discussion

Previously, some authors also studied the essential oil composition from the needles and twigs of* A. koreana*. Our result on the chemical composition of AEO is in agreement with previous studies. Similarly, Oh et al. [13] reported that limonene (23.5%), bornyl acetate (17.9%), *α*-pinene (11.1%), and camphene (10.2%) were the major components of essential oil from the needles of* A. koreana* (Table 2). The concentration of major components such as limonene (8.58–23.5%), bornyl acetate/bornyl ester (3.4–41.79%), camphene (10.2–22.5%), *α*-pinene (6.07–23.2%), and *β*-pinene (0.46–5.80%) was varied among the previously reported results [12, 13, 17–19]. Several authors have reported that the chemical composition of essential oil may differ according to the environmental (climatic, seasonal, and geographical), genetic differences, nutritional status of the plants, extraction methods, and analytical techniques [21, 22]. The AEO contains complex mixture consisting mainly of monoterpene hydrocarbons (60.93%). It is well known that monoterpenes are characteristic aroma components of various plants. Kim et al. [19] studied the memory enhancing effect of a supercritical carbon dioxide fluid extract of the needles of* A. koreana* on scopolamine-induced amnesia in mice. Further, the authors suggested that the essential oil of* A. koreana* showed a memory enhancing effect of 72.7% at 100 mg/kg and may be useful therapeutic agent against amnesia-inducing diseases.

In the recent decades, a number of studies have focused on the psychophysiological properties of aroma components using animal models. However, only few studies have been conducted to evaluate their efficacy in humans [23]. It is well known that the essential oils or aroma components provide a positive atmosphere by modulating the action of central nervous system. The EEG is a widely used neurophysiological evaluation technique to reflect the function of human brain. According to the frequency range, the EEG waves are categorized into five major EEG rhythms such as delta waves (0–4 Hz), theta waves (4–8 Hz), alpha waves (8–13 Hz), beta waves (13–30 Hz), and gamma waves (30–50 Hz). Many EEG studies have demonstrated significant alterations in the EEG spectrum values during the inhalation of aroma [5]. Furthermore, there has been extensive interest in the perceptual interaction between the two ears, two eyes, and two vestibular apparatuses. However, less consideration has been shown to the interaction between the two sides of the nose [24]. Hence, we examined the effect of inhalation of AEO on EEG activity of human in order to understand the influence of binasal and uninasal inhalations.

In the present study, the binasal inhalation of AEO shows significant increase of absolute alpha wave in left frontal and right parietal regions and absolute fast alpha wave in right parietal region (Table 3 and Figure 1). In addition, absolute mid beta waves increased in the same regions (left frontal and right parietal) and these changes might be enhancing the alertness state. The significant change of alpha 1 wave activity was observed after the inhalation of lavender oil, eugenol and chamomile [25]. Similarly, Iijima et al. [4] reported that the fast alpha activity significantly increased due to the inhalation of agarwood incense. Previous EEG studies on the influences of fragrances have demonstrated increased alpha wave activity by inhalation of various essential oils including lavender, sandalwood and chamomile. These aromatic oils have a relaxing effect on brain function. Further, the alpha wave activity is attenuated under emotional tension and stress states [3–5, 26]. In some extend, yoga increases relaxation state by increasing the frontal EEG alpha wave [27]. The present study clearly indicates that the increase of alpha wave activity due to the fragrance inhalation of AEO may contribute the brain functions in the form of mentally stable, increasing relaxation and feeling comfortable. In our previous study, the essential oil from the root of* I. helenium* produced significant changes in 7 indices during the inhalation. In particular, the reduction of absolute theta was observed in all the regions with the exception of left temporal region. In addition, absolute beta, absolute mid beta, and relative theta activities decreased during the inhalation of essential oil. These changes in the EEG activities may enhance the alertness state of the brain [28]. In another study, the relative high beta activity significantly increased in the right temporal region during the inhalation of (+)-limonene. On the other hand, the relative mid beta activity significantly decreased and the relative fast alpha activity significantly increased in the right prefrontal region during the inhalation of terpinolene [29]. From the results, it was observed that the fragrances of essential oils and their major components play a major role in the brain functions.

On the other hand, absolute beta and theta waves significantly decreased during the uninasal inhalation of AEO compared to before inhalation (Figure 1; Tables 4 and 5). In particular, the absolute beta waves such as low beta, mid beta, and high beta decreased mainly in the parietal regions during the inhalation through left nostril (Table 4). Lee et al. [30] stated that the low beta activity is mainly linked with drowsiness state, while high beta activity is mainly related to high awareness level. Therefore, uninasal (left) inhalation of AEO may increase the drowsiness state of brain function. The essential oil obtained from the leaves of* A. sibirica* reduced the arousal levels by increasing theta activity [31]. During performance of difficult task, the theta wave has been believed to maintain attention. In addition, reduction in theta wave activity is mainly related to the formation of memory [28, 32, 33]. In the present study, the reduction of theta wave activity suggests that the uninasal (right) inhalation of AEO may enhance the alertness and attention state of the brain function. The findings of the present study indicate that the fragrance inhalation of AEO essential oil highly affected the parietal and frontal regions than other regions.

In the present study, the binasal and uninasal inhalations of AEO showed different EEG power spectrum changes. In the uninasal inhalation, overall results revealed that the left nostril produced more EEG power spectrum changes than right nostril inhalation. Similar to our report, Herz et al. [34] suggested that the naming of odor was more accurate when odors were presented to the left nostril than to the right nostril. The variation in the EEG activities among the left and right nostrils may be due to the slight turbinate swelling in any one of the nostrils. Further, airflow difference between the nostrils causes each nostril to be optimally sensitized to different fragrances, so that each nostril communicates a slightly different olfactory image to the brain [7]. Hence, considerable variations were observed in detecting fragrances through uninasal inhalation [24]. Searleman et al. [35] suggested that each nostril projects information mostly to the ipsilateral hemisphere. While stimulation of the right and left nostrils will primarily affect the right and left hemispheres, respectively. In general, the two nostrils serve to smell different things altogether as a function of the nasal cycle. Gudziol et al. [36] found that 15% of the healthy subjects showed nostril side differences in the identification of odors. The healthy elderly subjects showed larger nostril side differences in the identification of odor when compared with younger subjects. Gudziol et al. [37] also suggested that the individuals with nostril side differences of olfactory function are at risk to develop bilateral olfactory loss within 4.5 years. Further, the slow yogic breathing through left nostril may alleviate stress and reduce cardiovascular disease risks compared to right nostril breathing [38].

In the gender difference, the AEO produced different EEG changes according to gender. During the inhalation of AEO, significant changes of EEG spectrum activities were observed mainly in women compared to men. The brains of men and women are differentially lateralized with respect to cognitive function. Previous reports have suggested that the gender differences exist in the EEG activity of resting, stimulus, and nonstimulus conditions [39, 40]. Doty and Cameron [41] extensively reviewed the influences of gender variation and reproductive hormone on human odor perception. Similar to earlier reports, the results of the present study also clearly indicated that the AEO showed different EEG power spectrum activities according to gender variation. In our recent study, the isomeric aroma components, (+)-limonene, and terpinolene produced different EEG activities in human according to gender difference [29]. From the results, the EEG spectrum activities highly affect men compared to women during both the binasal and uninasal inhalations of AEO. The results of the present study demonstrated the significance of AEO as psychophysiological stimulus, which produces different actions on brain function according to nostril and gender differences. Moreover, the binasal and uninasal inhalations of AEO also produced completely different actions on brain function.

The presence of various aroma components (D-limonene, bornyl acetate, camphene, *α*-pinene, and *β*-pinene) in the essential oil of* A. koreana* twigs might be responsible for the alterations of EEG spectrum activities and functions of human brain. Matsubara et al. [31] reported that essential oil of* A. sibirica* leaves significantly reduced the arousal levels after visual display terminal work. Further, the authors reported that *α*-pinene, santene, tricyclene, camphene, *β*-pinene, myrcene, *δ*-3-carene, limonene, and bornyl acetate were emitted from the essential oil of* A. sibirica* during the experiment. The essential oil from the needles of* A. sachalinensis* has anxiolytic effect and this oil mainly contains *α*-pinene, camphene, *β*-pinene, *β*-phellandrene, and bornyl acetate [42]. In aromatherapy, the essential oils obtained from the needles of* Abies* species have been commonly used for relaxation. According to the previous reports, it was observed that the essential oils from the various species of* Abies* contain almost similar profile of major components. In addition, one of the major components, *α*-pinene, has acetylcholinesterase inhibitory activity that is related to cognitive enhancement effect [19]. Another major component, limonene, highly influenced the human autonomic nervous system parameters and mental conditions [43]. Recently, Sugawara et al. [44] investigated the psychophysiological effect of 12 essential oils such as basil, bergamot, cardamom, cinnamon, juniper, lemon, orange, palmarosa, peppermint, sandalwood, spearmint, and ylang ylang. Based on the mental arithmetic and auditory tasks, the authors suggested that these essential oils may have versatile psychophysiological potencies.

## 5. Conclusion

The essential oil extracted from the twigs of* A. koreana* revealed the identification of 23 components including 13 hydrocarbons and 10 oxygen-containing compounds. Further, EEG power spectrum values changed significantly during both the binasal and uninasal inhalation of AEO. Based on the EEG changes, the binasal inhalation of AEO increases the relaxation and the uninasal (right) inhalation of AEO increases the alertness and attention states of human brain. However, uninasal inhalation of AEO through left nostril produces negative effect by increasing drowsiness state. Results of the present investigation provide evidence that the essential oil from the twigs of* A. koreana *could be used therapeutically for the positive psychological effects.

## Figures and Tables

**Table 1:** EEG power spectrum indicators used in this study.

S. number	Analysis Indicators	The full name of the EEG power spectrum indicators	Wavelength range (Hz)
1	AT	Absolute theta	4~8
2	AA	Absolute alpha	8~13
3	AB	Absolute beta	13~30
4	AG	Absolute gamma	30~50
5	ASA	Absolute slow alpha	8~11
6	AFA	Absolute fast alpha	11~13
7	ALB	Absolute low beta	12~15
8	AMB	Absolute mid beta	15~20
9	AHB	Absolute high beta	20~30
10	RT	Relative theta	(4~8)/(4~50)
11	RA	Relative alpha	(8~13)/(4~50)
12	RB	Relative beta	(13~30)/(4~50)
13	RG	Relative gamma	(30~50)/(4~50)
14	RSA	Relative slow alpha	(8~11)/(4~50)
15	RFA	Relative fast alpha	(11~13)/(4~50)
16	RLB	Relative low beta	(12~15)/(4~50)
17	RMB	Relative mid beta	(15~20)/(4~50)
18	RHB	Relative high beta	(20~30)/(4~50)
19	RST	Ratio of SMR to theta	(12~15)/(4~8)
20	RMT	Ratio of mid beta to theta	(15~20)/(4~8)
21	RSMT	Ratio of SMR~mid beta to theta	(12~20)/(4~8)
22	RAHB	Ratio of alpha to high beta	(8~13)/(20~30)
23	SEF50	Spectral edge frequency 50%	4~50
24	SEF60	Spectral edge frequency 90%	4~50
25	ASEF	Spectral edge frequency 50% of alpha	8~13

**Table 2:** Chemical composition of the essential oil from the twigs of *A. koreana*.

S. number	Component	RI^a^	RI^b^	Formula	Area%
	*Unsaturated terpene*				
1	Santene	888	902	C_9_H_14_	1.71 ± 0.72
	*Monoterpene hydrocarbons*				
2	Tricyclene	926	934	C_10_H_16_	1.96 ± 0.84
3	*α*-Pinene	939	946	C_10_H_16_	11.88 ± 0.85
4	Camphene	954	959	C_10_H_16_	12.48 ± 0.53
5	*β*-Pinene	979	980	C_10_H_16_	6.45 ± 0.36
6	3-Carene	1011	1013	C_10_H_16_	1.94 ± 0.22
7	d-Limonene	1021	1018	C_10_H_16_	25.29 ± 1.24
8	*γ*-Terpinene	1059	1064	C_10_H_16_	0.17 ± 0.05
9	4-Carene	1128	1131	C_10_H_16_	0.76 ± 0.05
	*Oxygenated monoterpenes*				
10	Linalyl formate	1216	1218	C_11_H_20_O_2_	0.43 ± 0.07
11	*β*-Fenchyl acetate	1232	1234	C_12_H_20_O	3.11 ± 0.20
12	Bornyl acetate	1285	1286	C_12_H_20_O_2_	19.31 ± 0.62
13	*α*-Terpinyl acetate	1349	1356	C_12_H_20_O_2_	1.95 ± 0.12
14	Geranyl acetate	1365	1368	C_12_H_20_O_2_	0.44 ± 0.06
	*Sesquiterpene hydrocarbons*				
15	Isoledene	1376	1380	C_15_H_24_	0.35 ± 0.07
16	Caryophyllene	1419	1412	C_15_H_24_	0.28 ± 0.03
17	*α*-Humulene	1454	1460	C_15_H_24_	0.18 ± 0.01
18	*γ*-Gurjunene	1477	1481	C_15_H_24_	1.73 ± 0.31
	*Oxygenated sesquiterpenes*				
19	Nerolidol	1563	1565	C_15_H_26_O	0.67 ± 0.07
20	Selina-6-en-4-ol	1624	1625	C_15_H_26_O	0.44 ± 0.07
21	*β*-Eudesmol	1650	1652	C_15_H_26_O	0.25 ± 0.14
22	Eudesm-7(11)-en-ol	1666	1670	C_15_H_26_O	5.38 ± 0.74
23	*α*-Bisabolol	1685	1689	C_15_H_26_O	1.36 ± 0.04
	Total				**98.52**

RI^a^: comparison of retention indices with those reported in the literature [20].

RI^b^: retention indices relative to *n*-alkanes (C_8_–C_20_) on the VF-5MS column.

**Table 3:** Effect of binasal inhalation of essential oil of *A. koreana* twigs on EEG activity in both genders.

EEG indices	Site	Before inhalation (*µ*V^2^)	During inhalation (*µ*V^2^)	*t*-test	*P* value^*∗*^
AA	F3: left frontal	61.112 ± 10.002	74.125 ± 13.246	−2.236	0.038
	P4: right parietal	66.517 ± 12.909	79.508 ± 15.743	−2.132	0.046
AMB	F3: left frontal	4.927 ± 0.456	6.141 ± 0.659	−2.359	0.029
	P4: right parietal	5.261 ± 0.556	6.833 ± 0.976	−2.357	0.029
AFA	P4: right parietal	15.023 ± 4.063	20.437 ± 6.167	−2.335	0.031
RMT	F3: left frontal	0.314 ± 0.023	0.366 ± 0.037	−2.249	0.037
	P4: right parietal	0.417 ± 0.040	0.554 ± 0.078	−2.944	0.008
RSMT	P4: right parietal	0.861 ± 0.073	1.048 ± 0.125	−2.357	0.029
RMB	P4: right parietal	0.067 ± 0.008	0.075 ± 0.009	−2.121	0.047
ASEF	P3: left parietal	10.178 ± 0.137	10.350 ± 0.153	−2.157	0.044
	P4: right parietal	10.250 ± 0.143	10.416 ± 0.166	−2.108	0.049

AA, absolute alpha; AMB, absolute mid beta; AFA, absolute fast alpha; RMT, ratio of mid beta to theta; RSMT, ratio of SMR~mid beta to theta; RMB, relative mid beta; ASEF, spectral edge frequency 50% of alpha.

^*∗*^Significant  difference (*P* < 0.05); number of subjects, 20.

**Table 4:** Effect of uninasal (left nostril) inhalation of essential oil of *A. koreana *twigs on EEG activity in both genders.

EEG indices	Site	Before inhalation (*µ*V^2^)	During inhalation (*µ*V^2^)	*t*-test	*P* value^*∗*^
AT	F4: right frontal	22.922 ± 2.728	18.246 ± 2.073	3.064	0.006
AB	Fp1: left prefrontal	15.552 ± 1.407	12.738 ± 1.087	2.117	0.048
	P3: left parietal	19.483 ± 2.115	16.379 ± 1.734	3.235	0.004
	P4: right parietal	16.967 ± 1.769	14.437 ± 1.636	2.612	0.017
ALB	P3: left parietal	7.634 ± 1.345	6.561 ± 1.086	2.140	0.046
AMB	P3: left parietal	7.981 ± 0.870	6.668 ± 0.742	2.399	0.027
	P4: right parietal	7.086 ± 0.808	5.822 ± 0.635	2.355	0.029
AHB	F4: right frontal	6.892 ± 0.646	6.048 ± 0.625	2.245	0.037
	P3: left parietal	8.430 ± 1.061	7.141 ± 0.970	3.196	0.005
	P4: right parietal	7.437 ± 0.901	6.368 ± 0.881	2.593	0.018
ASEF	T4: right temporal	10.106 ± 0.136	10.213 ± 0.133	−2.121	0.047

AT, absolute theta; AB, absolute beta; ALB, absolute low beta; AMB, absolute mid beta; AHB, absolute high beta; ASEF, spectral edge frequency 50% of alpha.

^*∗*^Significant difference (*P* < 0.05); number of subjects, 20.

**Table 5:** Effect of uninasal (right nostril) inhalation of essential oil of *A. koreana* twigs on EEG activity in both genders.

EEG indices	Site	Before inhalation (*µ*V^2^)	During inhalation (*µ*V^2^)	*t*-test	*P* value^*∗*^
AT	P3: left parietal	22.468 ± 3.501	18.520 ± 2.834	2.549	0.020
	P4: right parietal	19.540 ± 3.005	16.896 ± 2.696	2.580	0.018
RT	P4: right parietal	0.178 ± 0.017	0.158 ± 0.017	2.109	0.048

AT, absolute theta; RT, relative theta.

^*∗*^Significant  difference (*P* < 0.05); number of subjects, 20.

**Table 6:** Effect of inhalation of essential oil of *A. koreana* twigs on EEG activity in men (both, left, and right nostrils).

EEG indices	Site	Before inhalation (*µ*V^2^)	During inhalation (*µ*V^2^)	*t*-test	*P* value^*∗*^
*Both nostrils*					
RA	T3: left temporal	0.4271 ± 0.066	0.5103 ± 0.070	−2.593	0.029
	T4: right temporal	0.4273 ± 0.073	0.5066 ± 0.080	−2.459	0.036
RG	P3: left parietal	0.0998 ± 0.033	0.0805 ± 0.032	−2.400	0.040

*Left nostril*					
ALB	P3: left parietal	4.8147 ± 0.648	4.0104 ± 0.542	3.295	0.009
RLB	Fp1: left prefrontal	0.0326 ± 0.007	0.0437 ± 0.009	−3.819	0.004
	Fp2: right prefrontal	0.0334 ± 0.008	0.0438 ± 0.010	−2.824	0.020
	F3: left frontal	0.0370 ± 0.010	0.0433 ± 0.011	−2.394	0.40

*Right nostril*					
AT	F3: left frontal	22.2562 ± 5.108	18.1241 ± 3.968	2.330	0.045
	P4: right parietal	17.9657 ± 7.698	14.1190 ± 3.306	2.532	0.032

RA, relative alpha; RG, relative gamma; ALB, absolute low beta; RLB, relative low beta; AT, absolute theta.

^*∗*^Significant  difference (*P* < 0.05); number of subjects, 10.

**Table 7:** Effect of inhalation of essential oil of *A. koreana* twigs on EEG activity in women (both, left, and right nostrils).

EEG indices	Site	Before inhalation (*µ*V^2^)	During inhalation (*µ*V^2^)	*t*-test	*P* value^*∗*^
*Both nostrils*					
AFA	P4: right parietal	23.5915 ± 7.122	34.0754 ± 10.836	–2.635	0.027
RA	T3: left temporal	0.4515 ± 0.042	0.3999 ± 0.045	2.375	0.042
RT	P4: right parietal	0.1772 ± 0.028	0.1550 ± 0.033	2.519	0.033
AMB	T3: left temporal	7.4213 ± 1.660	9.6879 ± 1.575	–2.355	0.043
RFA	P4: right parietal	0.1930 ± 0.039	0.2245 ± 0.045	–2.526	0.032
RMB	T4: right temporal	0.0812 ± 0.008	0.1000 ± 0.014	–2.436	0.038
RSA	T3: left temporal	0.3525 ± 0.035	0.2955 ± 0.042	2.659	0.026

*Left nostril*					
AB	P3: left parietal	23.1918 ± 3.351	20.4436 ± 2.608	2.990	0.015
AG	P3: left parietal	6.6354 ± 1.433	5.6295 ± 1.228	2.371	0.042
	P4: right parietal	5.8777 ± 1.301	4.8122 ± 1.210	2.956	0.016
AHB	P3: left parietal	10.3107 ± 1.667	9.0508 ± 1.622	4.540	0.001
RT	F4: right frontal	0.2139 ± 0.028	0.1868 ± 0.024	3.441	0.007

*Right nostril*					
AMB	P4: right parietal	8.2244 ± 1.475	7.2582 ± 1.238	2.826	0.020
RT	F4: right frontal	0.2070 ± 0.019	0.1820 ± 0.018	2.305	0.047

AFA, absolute fast alpha; RA relative alpha; RT, relative theta; AMB, absolute mid beta; RFA, relative fast alpha; RMB, relative mid beta; RSA, relative slow alpha; AB, absolute beta; AG, absolute gamma; AHB, absolute high beta.

^*∗*^Significant difference (*P* < 0.05); number of subjects, 10.
